# Does the history of food energy units suggest a solution to "Calorie confusion"?

**DOI:** 10.1186/1475-2891-6-44

**Published:** 2007-12-17

**Authors:** James L Hargrove

**Affiliations:** 1 Department of Foods and Nutrition, University of Georgia, Athens, GA, 30602-0001, USA

## Abstract

The Calorie (kcal) of present U.S. food labels is similar to the original French definition of 1825. The original published source (now available on the internet) defined the Calorie as the quantity of heat needed to raise the temperature of 1 kg of water from 0 to 1°C. The Calorie originated in studies concerning fuel efficiency for the steam engine and had entered dictionaries by 1840. It was the only energy unit in English dictionaries available to W.O. Atwater in 1887 for his popular articles on food and tables of food composition. Therefore, the Calorie became the preferred unit of potential energy in nutrition science and dietetics, but was displaced when the joule, g-calorie and kcal were introduced. This article will explain the context in which Nicolas Clément-Desormes defined the original Calorie and the depth of his collaboration with Sadi Carnot. It will review the history of other energy units and show how the original Calorie was usurped during the period of international standardization. As a result, no form of the Calorie is recognized as an SI unit. It is untenable to continue to use the same word for different thermal units (g-calorie and kg-calorie) and to use different words for the same unit (Calorie and kcal). The only valid use of the Calorie is in common speech and public nutrition education. To avoid ongoing confusion, scientists should complete the transition to the joule and cease using kcal in any context.

## Introduction

The purposes of this article are 1) to note that the first known published definition of the Calorie from 1825 is available on the internet; 2) to suggest why W.O. Atwater selected the Calorie^a ^(modern kcal or 4.186 kJ) as a unit of potential energy for nutritional education and the first database of food composition; 3) to note the important connection between the man who defined the Calorie and Sadi Carnot; 4) to review the origin of other energy units; and 5) to explain how the kcal recently supplanted the Calorie even though both units had been obsolete since 1948. The article will conclude with a suggestion about how to eliminate the confusion that was caused because different scientific committees introduced disparate definitions for the same word.

## The dilemma of calorie confusion

Nutrition scientists, dietitians and clinical nutritionists face a dilemma that other scientists do not. Ever since the adoption of the international system (SI) of scientific units in the 1950's, the joule has been the only defined SI unit of energy. Neither the g-calorie nor the kcal is an SI unit. However, unlike other scientists, nutritionists are involved in public education concerning energy balance, and the U.S. lay public has been familiar with the Calorie for over 100 years. Indeed, the Calorie on U.S. food labels is one of the few tools available for public education about energy balance. At present, it is not helpful to ask lay people to set aside this tool and instead learn metric prefixes and SI terminology. An interesting but little known aspect of this situation is that the Calorie predated the joule by more than 60 years, and the original definition was almost exactly the same as presently found on U.S. food labels. One purpose of this article is to explain the priority of the Calorie relative to other energy units, and how it was displaced by the joule and kcal.

A second question that should be addressed is whether there is only one way to do away with the ambiguity imposed by using Calories, g-calories, and kcal in different contexts. This problem has even found its way into U.S. federal code. For example, in Title 21, section 104.20, part d of the Code of Federal Regulations[1], the following statements are made regarding food fortification (italics added):

"(1) A normal serving of the food contains at least 40 ***kilocalories ***that is, 2 percent of a daily intake of 2,000 ***kilocalories***;... (3) The food contains all of the following nutrients per 100 ***calories ***based on 2,000 ***calorie ***total intake as a daily standar..."

Clearly, if expert policy makers cannot use energy units consistently, there is little hope for public education. More than this, the present impasse was created by very convoluted historical events. Pragmatically, the Calorie is found on food labels because W.O. Atwater chose the unit to educate the lay public about food energy and also found it practical for compiling tables of food composition. Even though food databases have been updated to express energy as kcal and kJ, food labels have not changed because they are primarily used for education of the lay public, who may only have grade school education. Therefore, confusion of names for food energy primarily affects nutrition educators in clinical and private practice, rather than scientists who can be expected to understand SI units. Let us examine how the present situation arose.

## Definitions of energy units

Before discussing the history of the energy units that are commonly used in nutrition, it is useful to provide their present definitions:

### Joule

The joule (J) is the only unit of energy defined in the SI system. It is the work done by a force of 1 newton (N) moving an object 1 meter in the direction of the force, and has base units of kg m^2 ^s^-2^. These units can be derived as the work of lifting a mass (kg) a distance (m) against gravitational acceleration at sea level (9.806 m s^-2^). Because the work of lifting 1 kg by 1 m equals 9.8 J, 1 J is the work done in lifting 0.102 kg by 1 m. Alternatively, it is the work required to move an electric charge of 1 coulomb through an electrical potential difference of 1 volt. It can also be defined as the work done to produce power of 1 watt continuously for 1 second (1 W-s). Note that these definitions are stated in terms of work but none relates directly to heat or potential energy.

### calorie

The "small calorie" or "g-calorie" is defined as the amount of heat required to raise the temperature of 1 g of water by 1°C with a temperature change from 14.5 to 15.5°C. The current US Dietary Reference Intakes define 1 cal as 4.186 J [2]. Some texts use the thermochemical calorie, 4.184 J.

### Calorie

The Calorie was originally defined as the amount of heat required to raise the temperature of 1 kg of water from 0 to 1°C at 1 atmosphere of pressure. When used to express potential energy on food labels, it is defined as 4.186 kJ and is identical to a kcal.

### kilocalorie

When the m-kg-s system was adopted in the 1930's, the cal was defined as a thermal unit in the cm-g-s system and 1 kcal was defined as 1000 cal in the m-kg-s system. All forms of the calorie were deemed obsolete in science after the SI system was adopted in the 1950's.

It is worthwhile to note that the Calorie (or kcal) has consistently been used to indicate the potential energy in foods (or the chemical energy stored in human tissues), whereas the primary use of the joule is as a unit of work or of energy in general.

## Why did W.O. Atwater choose the Calorie?

**Figure 1:**
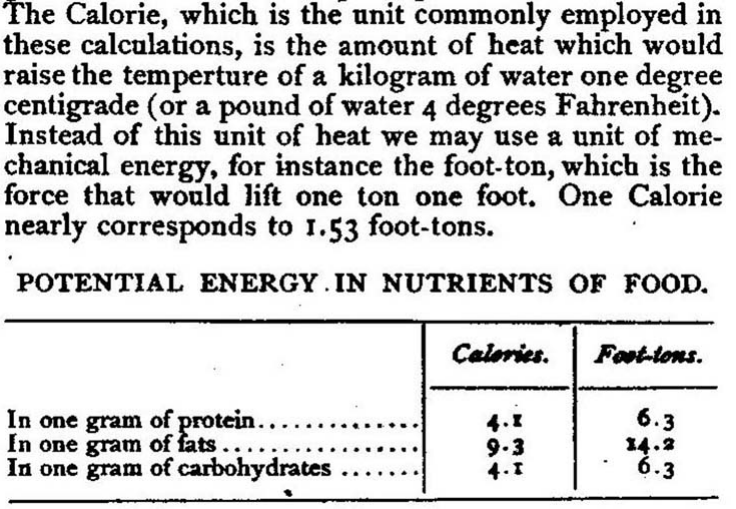
W.O. Atwater introduced the Calorie to US audiences in an 1887 article in Century magazine. Note that he defined the Calorie as potential energy needed to support a given amount of physical work against gravity, which he calculated as foot-tons.

The U.S. public began using the Calorie only after W.O. Atwater introduced it as an energy unit for foods in 1887. His series of 5 articles on food constituents was published in a popular periodical called *Century *magazine and was addressed to the educated lay public. Because his article on food energy (*The Potential Energy of Food *[3]) is a milestone in nutritional science but is out of print, a facsimile has been appended to the present manuscript [See Additional file 1]. As shown in Fig. 1, Atwater not only defined the Calorie in terms of heating 1 kg of water, he also indicated that it was the amount of potential energy required to perform about 1.53 foot-tons of physical work at perfect efficiency. The modern equivalent is 426.6 kg-m at 1 *g *(4,186 J). On a following page, he noted that the mechanical efficiency of machines was about 8% but that of humans and animals was about 20–30%. In short, the central point of his article was to explain the need to provide sufficient potential or chemical energy from food to support manual labor.

**Figure 2:**
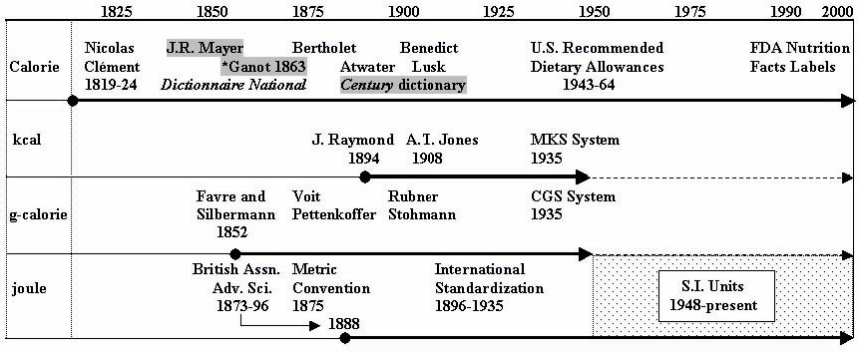
Timeline comparing use of the Calorie (kg basis or kcal), g-cal, kcal and joule. MKS, m-kg-s system of units; CGS, cm-g-s system of units. First known usage of the Calorie in German was by Mayer in 1845 [29]. First known occurrence in English was when Adolphe Ganot's physics text [6] was translated in 1863.

**Figure 3:**
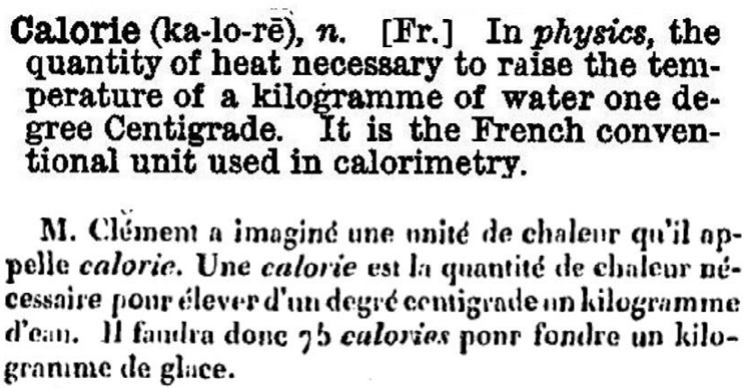
Top, the calorie as defined in the 1883 *Imperial Dictionary [7]*. Bottom, the original published definition of the calorie as described in *Le Producteur *(1825) [19].

As will be shown below, the Calorie was the only named energy unit that existed in English dictionaries of the time (Figs. 2 and 3). The joule had been proposed as an electrical unit in 1882, but had not entered the lexicon, and the small or g-calorie was not defined in dictionaries (it was used in some scientific papers, however). This record can be verified by consulting the Oxford English Dictionary [4], which identifies word origins. As a scientist, however, Atwater was certainly aware of the g-calorie and may have known that a commission of the British Association for Advancement of Science was discussing alternative units. The reason that he chose the Calorie lay in his purpose of educating the public about rational food choices.

In the sense of being a unit of heat, the word *calorie *did not enter the English language until the 1860's [5]. Earlier editions of Webster's dictionary defined *calorie *as "a principle of fire or heat." This usage suggests that before *calorie *became a heat unit, it might have been an alternative for, or confused with, caloric (*calorique*), which entered English usage in about 1891 (OED). Usage changed when Adolphe Ganot's French physics text was translated into English in 1863[6]. The Calorie then entered the English vocabulary with the same definition that French physicists used. Thus, the 1883 edition of the English *Imperial Dictionary *[7] defined the calorie as "the quantity of heat necessary to raise the temperature of a kilogramme of water one degree Centigrade" (Fig. 3). Ganot specified that the initial temperature was 0°C (implicitly assuming a pressure of 1 atmosphere).

In the 19^th ^century, a large portion of most workers' income was spent on food for family members and livestock. When asked to write his articles in *Century *magazine, Atwater had just returned from post-doctoral studies in Karl Voit's laboratory where he also worked with Max Rubner[8]. In non-English speaking countries, the g-calorie was the customary unit of calorimetry because most scientists believed that Favre and Silbermann [3] had invented the calorie and defined it in that sense. After the relationship between heat and work was established, many workers alternatively used the kg-m or ft-lb as units, or else defined unnamed "heat units" in reference to changes in water temperature. Even 20^th ^century histories and biographies mistakenly credit Favre with naming the calorie[9,10]. Voit and Rubner used the g-calorie or the German term for unnamed "heat units." Although it seems puzzling that Atwater would have switched from the g-calorie to the Calorie, there is probably a simple reason.

The Century Company published both *Century *magazine and W.D. Whitney's comprehensive *Century Dictionary *(later, the *New Century Dictionary*). It would have been natural for Atwater's editor to verify that the Calorie was defined in the dictionary that was published by his own firm. In that dictionary, the Calorie was defined as the heat needed to raise the temperature of 1 kg of water from 0 to 1° centigrade. There was no entry for a g-calorie or a joule. As an educator, Atwater would have realized that the g-calorie was too small because over 2 million units would be needed per day. Moreover, the lay public was not familiar with metric prefixes and it would have been unnecessarily complex to add the kilo-prefix. The Calorie was already defined based on the heat capacity of 1 kg of water. However, in the 1870's, French chemist Marcellin Berthelot had observed that there were two definitions for the same word. He decided to define the lower-case *calorie *as a g-calorie and use the capitalized Calorie to refer to the kg-calorie[11]. Later dictionaries adopted this custom and began referring to "small" and "large" calories. In contrast, the kilocalorie was not introduced as a heat unit until 1894–1908[12,13]. Therefore, when Atwater's series on nutrition was published, the energy units available were the large Calorie, the small calorie, or work units of kg-m and ft-lb. He chose the Calorie (with equivalent ft-tons) and the public quickly accepted the new word. Moreover, because food energy was expressed as Calories in subsequent food tables [14,15], nutrition science likewise adopted this unit for energy. The happy state in which American nutrition scientists, educators, and the public used one definition lasted from 1887 until about 1970. The question of origins then becomes why early French dictionaries and physicists such as Ganot[6] had defined the Calorie in terms of heating a kg of water, rather than the small or g-calorie that was used by Favre and Silvermann [16]. How did the Calorie enter the French lexicon?

## Coinage of the Calorie as a unit of heat

Although some authors have suggested that Lavoisier named the calorie [17], Ziegler notes that the word was never used in his original publications[18]. The first known published definition of the calorie (1825) occurred in a Parisian journal called *Le Producteur. Journal de l'Industrie, des Sciences et des Beaux-Arts *[19]. (The quotation may be found on the Gallica Internet site [20] by doing a title search for *Le Producteur *and then entering 583 in the search box, *Aller Page*.)^b ^A portion of that page is reproduced in Fig. 3, which comes from a very detailed series of anonymous articles that describe a course on industrial chemistry given annually by Professor Nicolas Clément-Desormes^c^. Much of the course discussed the theory by which steam engines convert heat into useful work, and a unit of heat was required. The first step in calculating fuel efficiency was to define how much energy is contained in fuels. Therefore, in discussing calorimetry, Clément provided a definition that was recorded by an anonymous auditor, translated as:

Clément imagines a unit of heat that he names *calorie*. One *calorie *is the amount of heat needed to elevate by one degree centigrade one kg of water.

Many prominent people enrolled in Clément's course during the decade that he taught it, and two sets of course notes survive[21]. The first hand-written definition of the calorie is in J.M. Baudot's notes of 1824 [22]. Clément provided the first technical definition of the Calorie, and it was significant for two reasons. Firstly, he defined the unit specifically in terms of heating 1 kg of water from 0 to 1°C. More importantly, his definition was accepted by engineers of the period and made its way into French dictionaries and physics texts by the 1840's[5,18]. The original source of confusion concerning the origin of the Calorie may be ascribed to the lack of a published definition in a scientific journal, and the failure of most scientists to consult dictionaries! This problem was compounded by the absence of any international system of energy units until after the Metric Convention of 1875[23].

## Clément and Carnot calculate a mechanical equivalent of heat

Nicolas Clément was a noted professor and industrial chemist[24] with many interests besides the theory of heat. He had trained at the *École Polytechnique *with Charles Desormes, who was an assistant in the laboratory of Guyton de Morveau, a renowned chemist and colleague of Lavoisier, Berthollet, and Forcroy[25]. From 1801–1819, Clément and Desormes published numerous papers on topics such as the composition of carbon monoxide, proof that iodine is an element, a value for absolute zero, and a value for the ratio of the specific heats of gases at constant pressure and constant volume that is called γ [24]. The value of γ is important because it provided a means of calculating the mechanical equivalent of heat (Joule's coefficient) [26].

From 1812–1819, Clément and Desormes conducted studies on the nature of heat and derived an algebraic method for calculating the mechanical power that can be obtained from steam. Clément read the paper to the *Académie des Sciences *in August, 1819, more than 20 years before Mayer or Joule took up this subject. Parts of the manuscript were published in the *Bulletin de la Societe' d'Encouragement *in 1819 and later donated to the Royal Society of London[27]. The method for calculating mechanical power was sometimes called the Law of Clément-Desormes. Fox[21,28] and Lervig[22] state that two key concepts in the paper were the conservation of heat (*calorique*) and adiabatic (rather than isothermal) expansion of steam vapor.

The record shows that Clément not only defined the Calorie but also could calculate the amount of work that could be obtained from steam. Clément taught his students that the energy content of charcoal was 7050 Calories (kcal) per kg, and that 650 Calories was required to convert 1 kg of water to steam. One kg of water vapor could do work as it expanded from 1 L to 1700 L. Clément assumed conservation of energy (or *calorique*) and employed engineering units for work (*Dynamie*) equivalent to lifting 1000 kg to a height of 1 m. Clément noted that steam engines of the day could obtain about 300,000–400,000 kg-m of work from 1 kg of charcoal. Without considering efficiency, this would give a value of less than 57 kg-m/kcal. This is about 13% of the theoretical maximum, but Clément probably did not have a way of calculating absolute thermodynamic efficiency. One of Clément's important contributions was to show that higher operating temperatures and pressures permitted greater efficiencies.

Around 1819, Clément was introduced to Sadi Carnot and gave him a copy of his paper on the motive power of steam. Carnot clearly thought that Clément's approach was not fully satisfactory, and derived an alternative equation with 3 parts that correspond to a production phase, expansion, and release of spent steam. Carnot later gave his colleague an unpublished manuscript, "Recherche d'une formule propre à représenter la puissance motrice de la vapeur d'eau." One equation for motive power, F, was written as follows[28].

F=N∗ln⁡pp'(267+t+t'2)
 MathType@MTEF@5@5@+=feaafiart1ev1aaatCvAUfKttLearuWrP9MDH5MBPbIqV92AaeXatLxBI9gBaebbnrfifHhDYfgasaacPC6xNi=xI8qiVKYPFjYdHaVhbbf9v8qqaqFr0xc9vqFj0dXdbba91qpepeI8k8fiI+fsY=rqGqVepae9pg0db9vqaiVgFr0xfr=xfr=xc9adbaqaaeGacaGaaiaabeqaaeqabiWaaaGcbaGaemOrayKaeyypa0JaemOta4Kaey4fIOIagiiBaWMaeiOBa4wcfa4aaSaaaeaacqWGWbaCaeaacqWGWbaCcqGGNaWjaaGccqGGOaakcqaIYaGmcqaI2aGncqaI3aWncqGHRaWkjuaGdaWcaaqaaiabdsha0jabgUcaRiabdsha0jabcEcaNaqaaiabikdaYaaakiabcMcaPaaa@4316@

N equals 48.2, and is the ratio P*V/367 where P is the pressure of a 10.4 m column of water and V is 1700 L (the volume of 1 kg of steam). P, p', t and t', respectively, are the vapor pressures and temperatures at the beginning and end of the cycle of operation. Carnot gave an example with p = 760 mm Hg, p' = 9.47 mm Hg, t = 100°C and t' = 10°C. He reported a value of 66,278.5 kg-m but rounded the number to 66,000 because he regarded it as imprecise. Fox[28] states that the correct value of F under these conditions is 66,734.8. By dividing this work by the number of Calories required to heat the kg of water to form steam, the result is 66278/650 = 102 kg-m/Cal.

The maximum amount of work that can be obtained from a perfectly efficient machine using 650 Calories of fuel is found by multiplying the number of Calories times Joule's coefficient, 427 kg-m/kcal. The answer is 277,550 kg-m. This indicates that Carnot's equation gave an answer that represents 23.8% efficiency. Probably, the calculated maximum is not equal to the ideal because perfect efficiency is only obtained if the condenser is operating at absolute zero. William Thompson (Lord Kelvin) later calculated Carnot cycle efficiency from the equation,

η=(To−Tc)To
 MathType@MTEF@5@5@+=feaafiart1ev1aaatCvAUfKttLearuWrP9MDH5MBPbIqV92AaeXatLxBI9gBaebbnrfifHhDYfgasaacPC6xNi=xI8qiVKYPFjYdHaVhbbf9v8qqaqFr0xc9vqFj0dXdbba91qpepeI8k8fiI+fsY=rqGqVepae9pg0db9vqaiVgFr0xfr=xfr=xc9adbaqaaeGacaGaaiaabeqaaeqabiWaaaGcbaacciGae83TdGMaeyypa0tcfa4aaSaaaeaacqGGOaakcqWGubavdaWgaaqaaiabd+gaVbqabaGaeyOeI0Iaemivaq1aaSbaaeaacqWGJbWyaeqaaiabcMcaPaqaaiabdsfaunaaBaaabaGaem4Ba8gabeaaaaaaaa@3A2A@

where η is efficiency, T_o _is operating temperature, and T_c _is condenser temperature (K). At the temperatures stated, efficiency equals 23.5%. This correction would yield a mechanical equivalent of heat equal to 422 kg-m/kcal, which is very close to the modern value. The manuscript that Carnot provided to Clément does not discuss what later became known as Joule's coefficient or the mechanical equivalent of heat. However, Carnot did write a note to himself ([28], p. 191) that "the production of one unit of motive power requires the destruction of 2.70 units of heat." This indicates that he had calculated that 1 Calorie was equivalent to 370 kg-m of work (1000/2.7). This was the same value that Mayer later found, presumably because both men calculated the equivalence using the gas law (the logic is explained in[26], pp. 107–9). Mayer is also the first man known to have used the Calorie in a German publication[29], and he recommended using the kg-m as a common unit of work and energy

The recovered manuscripts demonstrate that the man who invented the Calorie was thinking deeply about heat. He not only defined a Calorie and used it in his calculations, but understood that the energy in fuels was related quantitatively to the amount of work that could be obtained from a heat engine. Clément and Desormes had developed an algebraic method of calculating how much work could be obtained from a steam engine as a function of the temperature and pressure of the piston and the condenser. Carnot solved the same problem using integral calculus and gave Clément a copy of his formula as well as his paper describing what is now called the Carnot cycle. Evidence suggests that Carnot knew that a "mechanical equivalent of heat" existed, but there is no record that he told Clément how to calculate the theoretical maximum. It is stunning that work of this prescience was not published and remained unknown to anyone who had not taken Clément's course. Through his influence on other chemists and engineers, it seems very likely that Nicolas Clément was indirectly responsible for the Calorie entering the French lexicon [30]. However, with no publication other than dictionaries to cite, the origin of the Calorie was unknown to Atwater and other scientists who later used the term.

## Caloric equivalent of work by humans and animals

Whereas Clément was interested in obtaining maximum work from a given amount of fuel in industrial settings, Atwater's nutritional studies were motivated by a desire to provide nutritious yet inexpensive food for people who were accustomed to physical work. In explaining the Calorie, he indicated that the heat unit was equivalent to about 1.53 foot-tons mechanical energy (Fig. 1). He also noted that human mechanical efficiency was about 20%. Let us derive the modern "mechanical equivalent" in relation to kJ and kcal.

The work (J, with base units of kg m^2 ^s^-2^) done in lifting a mass (m) of 1 kg by a height (h) of 1 m against gravity (*g*) is:

W = m*h*g = 1 kg*1 m*9.806 m s^-2 ^= 9.806 J

1 kJ = 102 kg-m (at 1 *g*)

1 kcal = 102 kg-m/kJ*4.186 kJ/kcal = 427 kg-m

The kg-m unit employs the kg as force (like the English or US pound) rather than mass because it assumes that the mass is being acted on by gravitation at sea level. If one converts 427 kg-m to US units, it is equivalent to about 3100 ft-lb or 1.55 ft-tons. These considerations lead to a very simple way of estimating the energy needed to climb. If stated in units that are relevant in nutrition (kJ), it is:

W=m∗h∗g∗10−3e=m∗h102∗e
 MathType@MTEF@5@5@+=feaafiart1ev1aaatCvAUfKttLearuWrP9MDH5MBPbIqV92AaeXatLxBI9gBaebbnrfifHhDYfgasaacPC6xNi=xI8qiVKYPFjYdHaVhbbf9v8qqaqFr0xc9vqFj0dXdbba91qpepeI8k8fiI+fsY=rqGqVepae9pg0db9vqaiVgFr0xfr=xfr=xc9adbaqaaeGacaGaaiaabeqaaeqabiWaaaGcbaGaee4vaCLaeyypa0tcfa4aaSaaaeaacqWGTbqBcqGHxiIkcqWGObaAcqGHxiIkcqWGNbWzcqGHxiIkcqaIXaqmcqaIWaamdaahaaqabeaacqGHsislcqaIZaWmaaaabaGaemyzaugaaOGaeyypa0tcfa4aaSaaaeaacqWGTbqBcqGHxiIkcqWGObaAaeaacqaIXaqmcqaIWaamcqaIYaGmcqGHxiIkcqWGLbqzaaaaaa@4579@

Where W is kJ of energy, m is mass (kg), h is height (m), 10^-3 ^converts J to kJ, e is efficiency (unitless, a value between 0.2 and 0.3), and 102 is the mechanical equivalent (kg-m/kJ) (Eq. 4). If one prefers to express energy as kcal, the mechanical equivalent is 427 kg-m/kcal (Eq. 5). Eq. 6 may appear unfamiliar, but it is the original form of the energy balance equation that relates the potential energy in food to the expenditure of mechanical energy that the food will support. The equation implies that one who consumes 1 kcal must perform work equal to some fraction of 427 kg-m to remain in energy balance. For example, how high a mountain would a 70 kg person need to ascend to eliminate the energy in 1 pound (0.454 kg) of fat? If one assumes the fat contains 3500 kcal of energy and that efficiency is 20%, the answer is about 4,270 m (14,000 feet, similar to Mt. Rainier). This relationship was self-evident to Atwater but has been lost from modern nutrition texts.

## Advent of the g-calorie

From 1824 to 1851, several well-known dictionaries and physics texts defined the Calorie in terms of heating 1 kg of water[6,18,31]. Despite this, the definition must not have been widely known among chemists. Favre and Silbermann published a series of studies on heats of oxidation of acids and bases, and in 1852 [16] used the calorie as a heat unit based on a mass of 1 gram. Whereas Clément never published his definition outside of *Le Producteur*, Favre and Silbermann published extensively in prominent chemical journals and their work was well known in the field of chemical calorimetry. Perhaps because there was no way to cite Clément's work, most scientists assumed that Favre had invented the unit and that it was defined as a g-calorie. Nonetheless, the large Calorie was still the only unit defined in dictionaries and physics texts.

Because of the dual origins of the calorie, by the 1860's, the same word was being employed in reference to g-calories and kg-calories, but no one had applied metric prefixes to the units. Finally in 1879, the chemist, Marcellin Berthelot, differentiated the two units by capitalizing the large or kg-calorie and noting that it equaled 1000 of the smaller g-calories [11]. Prior to that time, the calorie was most often written with lower case units, but because the word is a noun, it was capitalized when written in German. Certainly, Berthelot knew the metric prefixes and it is unclear why he did not apply them to solve the problem. A timeline of these events is shown in Fig. 2.

Karl Voit was a prominent German physiologist who developed one of the first laboratories that could evaluate food energy and human energy usage. He certainly would have known of Mayer's work on the caloric equivalence of physical labor, but did not adopt the kg-calorie as a standard. Instead, Voit began using the g-calorie in lectures on human calorimetry in 1866, and stated that daily metabolism of one male subject was 2.25 to 2.4 × 10^6 ^g-calories, depending on prior diet[32], p. 35. The most likely reason for this choice of energy units was that students of calorimetry knew about Favre and Silbermann's work but not Clément's. By 1883–85, Voit's student, Max Rubner, had published papers using the g-calorie to define heats of combustion for food and heat produced in respiration studies [33-35]. In the same period, Henneberg and Stohmann were using calorimetry for proximate analysis of livestock feeds at the Weende Experiment Station [36].

## Debut of the kilocalorie

It is not known what source Raymond used when he named the kilocalorie in his 1894 textbook of medical physiology[13], but the unit was not in general use. He may simply have decided to avoid confusion between g-calories and kg-calories by using accepted metric prefixes. Even in 1903, English dictionaries still defined the calorie relative to a kg of water and there was no definition of a kcal. The kilocalorie was not introduced in an indexed scientific publication until after Armsby proposed a new energy unit to be called a Therm in 1907[37]. In response, A.T. Jones wrote a letter to the editor of *Science *noting that Armsby's new name was unnecessary, and reminded readers of the convention of using metric prefixes. Jones specifically stated that if one accepted the g-calorie as a unit in the cm-g-s system, then the next larger energy unit should be called a kilocalorie[12]. In 1909, a "kilocalory" was introduced in a supplement to the *New Century Dictionary*. The kcal began to enter other dictionaries after the m-kg-s system was introduced in the period between 1918–1935[38,39]. The OED notes that by 1923, European physics texts were employing the kilocalorie as a unit of heat and that the unit was known to German law. In 1927, the *New Century Dictionary *still defined the "calory" as the heat needed to raise the temperature of 1 kg of water by 1°C, but noted that a "small calory" based on heating 1 g of water was also used. Beginning in about 1935, the kilocalorie began appearing in English dictionaries and the period of "calorie confusion" set in.

## Origin of the cm-g-s system and the joule

The base units in the original metric system were the meter, the kilogram and the second. Admittedly, the way the units are named suggests that the g is the base unit and the kg is a derived unit. However, the metric system was originally intended for commerce and the kg was based on the weight (not mass) of 1 liter of water at 0°C. Energy was not considered to be an item of commerce, and no units of energy were suggested by any official organization until the First Law of Thermodynamics was understood. A commission was established by the British Association for Advancement of Science after 1862 to define precise electrical units. The members of the commission were primarily British physicists and engineers; no member of the committee had a background related to nutrition. They introduced the cm-g-s system in 1873 and named the dyne and the erg as units of force and work[40]. They then departed from the rule of naming units with Greek or Latin roots and decided to honor important scientists such as Ampere, Ohm, Volta and their colleagues, Watt and Joule. The OED states that the joule was proposed by Siemens in 1882 and the British Association adopted it for the cm-g-s system in 1888[41]. The joule was originally an electrical unit, but the committee realized that the same unit could be used for heat, work, and any other form of energy. By 1896, the committee had decided that a g-calorie could be considered a secondary unit of energy[41,42]. The committee noted that there was no agreement concerning what temperature of water should be selected as a basis for defining the calorie. It seems evident that no committee member thought to check the definition of calorie in the *Imperial Dictionary*[7], nor did anyone observe that it would have been satisfactory to leave the unit at 0°C and make corrections based on tables of the specific heat of water as a function of temperature [43]. It would also have been feasible to base the Calorie on the molar heat of benzoic acid, as was normal practice in calorimetric studies [26].

The units of the cm-g-s system were too small for many scientists, and it seems that the use of metric prefixes to define different scales was not an automatic standard. To accommodate a larger scale, the m-kg-s system was proposed around 1918[38]. Therefore, prior to the development of the *Systém International des Unites*, the m-kg-s system and cm-g-s system co-existed (Fig. 2). In 1935, the International Electrotechnical Commission adopted the m-kg-s system. It accepted the g-calorie as a thermal unit in the cm-g-s system and the kcal for the m-kg-s system[39].

The Bureau International des Poids et Mesures (BIPM) was established in 1875 to reach consensus on basic metric units. During the 1930's, the BIPM convened the Consultative Committee on Thermometry (CCT) to clarify standards of heat. The committee was led by W.H. Keesom, who summarized a proposition that the calorie should equal 1/860 watt-hours or 3600/860 joules (4.186 J)[44]. From then on, any secondary thermal unit was to be defined relative to the joule rather than to the heating of water at any temperature. The 1948 General Conference also recommended discarding the calorie because it could not be derived directly from basic units. In 1954 the SI base units were adopted, and in 1970, the Committee on Nomenclature of the American Institute of Nutrition advised that the kilocalorie should be replaced by the kilojoule (kJ) in scientific publications[45,46].

From 1935 forward, most scientists probably believed that the g-calorie had been a base unit in the original metric system. Clément's definition had been entirely forgotten, and no one seems to have objected that the Calorie had been defined differently in dictionaries for 50–100 years. It did not matter to the physicists and electrical engineers that ordinary people who used food tables had never heard of the joule. Nutrition scientists may have noted Kennelly's article on the m-kg-s system[39], but the Calorie had been the nutritional unit of potential energy since the first food tables were published[3,8,14] and no one was in a rush to change.

## The transition from Calories to kcal in nutrition

By the time the kcal became a recognized unit, the venerable Calorie had been in the U.S. English lexicon for over 50 years. Because the Calorie was adopted as a unit to express the physiological fuel values of foods in USDA Farmers' Bulletins[14,15], the unit made its way into articles and books that dealt with weight reduction. For example, Dr. Lulu Hunt Peters' popular "Diet and Health with Key to the Calories" specifically cited Farmers' Bulletin 142 as a source of information [47]. Because of similar precedents, nutrition-related publications worldwide employed the Calorie as the sole energy unit until about 1960. This usage became dominant when the U.S. Recommended Dietary Allowances (RDA) began to employ the Calorie from 1943 until 1956[48]. The Calorie was also used in handbooks for clinical dietitians and medical practitioners[49]. Ironically, as S.I. units developed after 1948, it was recommended that all forms of the calorie be abandoned. Beginning in 1960, papers published in nutrition and dietetics sometimes noted that the Calorie was the same as the kcal, and the same point was made in the 1964 U.S. RDA. By 1968, the kcal had replaced the Calorie as the unit of choice in the RDA, and between 1964–1970, most nutrition journals made the transition. It is ironic that by 1954, the physicists and engineers who had instigated the change had abandoned calories in favor of the joule. To the extent that the kcal was ever an "official" unit, its reign only lasted from 1935 to 1948! Nevertheless, editorial style guides for essentially all international journals in the life sciences accepted the g-calorie as a base unit and allowed the kcal to be employed to express larger quantities of energy.

The outcome of the attempt to become more sophisticated about energy units was that a word that was understood by scientists and the public alike from 1887 until about 1960 was abandoned by the professionals. From 1970 on, all nutrition texts had to change from Calories to kcal, although some protests were made. The chapter on energy in Goodhart and Shils' 1980 *Modern Nutrition in Health and Disease *[50] defined the Calorie, the kcal and the joule but complained, "Personally, I am happy with calories." One can easily verify the late transition from Calories to kcal by checking back issues of *AJCN*, the *Journal of Nutrition *and any nutrition textbooks published before 1975. This history is probably unknown to any scientist who earned a doctorate after that year.

## Summary

History shows that the Calorie of food labels has priority over other energy units, dating to at least 1824. Scientists of the time were using calorimeters in a discipline called calorimetry, and it was quite natural to adopt a heat unit with the same Latin root. Existing manuscripts show that the unit was initially defined in Clément's course on industrial chemistry [22] and a published description of the course [19], and not in a publication that was recognized by scientific bodies. One lesson that any scientist would draw from this history is that it is essential to publish ideas and definitions in peer-reviewed journals. Although Nicolas Clément did not do this, his influence was sufficient that the word gained a dictionary definition by the 1840's. The Calorie was defined in engineering publications during the 1820's, and it is inexcusable that later workers failed to note these sources [30].

Arguably, the Calorie is the energy unit that is best understood by the US public. In contrast, the pedigree of the g-calorie began in 1852, and the kcal in 1894–1908. The joule was developed as an electrical unit and a common unit of energy in the 1880's. After the Calorie was adopted by Atwater in 1887, it was used consistently and without confusion in nutrition science until 1964–70. Oddly, when the kcal was introduced as a nutritional unit beginning with the 1964 U.S. RDA, it had already been superseded because S.I. units had replaced the awkward division into cm-g-s and m-kg-s systems. However, most scientific journals adopted the 1935 proposal to allow the joule, the calorie (former g-calorie) or the kcal and eliminate the Calorie. Current style guides show that this is still the case, even though the kcal of the m-kg-s system and g-calorie of the cm-g-s system are officially obsolete. In contrast to U.S. food labels, European food labels must list kJ and kcal. Both units are also reported in USDA databases.

The history discussed here explains why the Calorie came to be used not only in food databases and on nutrition labels but also in most popular recipe books that include nutrition information. One can easily verify that the Calorie (capitalized or not) is the most common unit of food energy found in recipes and articles on the Internet. It is interesting that there is little confusion of usage in popular culture except after scientists helpfully try to explain that the "proper" term is kcal and that it rightly should be converted to kJ. This leads one to wonder whether the physicists and chemists who multiplied the energy units ever took a lunch break.

The motivation for writing this article was to show why W.O. Atwater chose the Calorie (modern kcal) instead of the g-calorie and to explain more about the contributions of Nicolas Clément-Desormes^c^, the man who probably invented the calorie. The Calorie has a 140 year pedigree in the English language, and there is a question of whether it can or should be dislodged by academics and policy makers. It is a practical unit that lacks the pretension of metric prefixes. It was coined using good rules of naming because *calor *means heat. The definition is familiar to anyone who has ever heated water. Moreover, energy needs are easy to calculate because men need about 100 Calories an hour, and women somewhat fewer. None of this is true if kJ are substituted. Most U.S. nutrition educators probably would agree that Dr. Atwater made an excellent choice.

## Recommendations

Firstly, educated people including college students should understand that all forms of energy can, in principle, be interconverted according to the First Law of Thermodynamics. Because of this, it is convenient to employ one common energy unit (the joule) that enables scientists and engineers to communicate freely using the SI system. It is true that a number of obsolete energy units such as the erg and the therm have ceased to be used in most areas of science. However, there are still reasons to employ different energy units in special circumstances. Good examples are cases such as the electron volt and hartree in which differences in scale make it cumbersome to employ the joule. The fact that it is preferable to use the joule does not mean that it is always the best choice. One might argue that the Calorie has always been used as a unit of potential energy in food and the equivalent energy in human tissues that can be removed by work, rather than work itself (J).

Secondly, if it be agreed that the joule should be the common unit of energy and work for scientific correspondence, then it would follow that journals should stop permitting the obsolete kcal to be used as an energy unit. With that unit discarded, there would no longer be a need for the small calorie to be used in any context, thereby eliminating the major point of ambiguity with common English usage. Furthermore, nutritional databases could remove the kcal and continue to employ kJ. This action would eliminate the problem of using two words (kilocalorie and Calorie) for the same quantity of heat. The simplest intermediate course would be to recognize that the Calorie is an accepted English word and include it along with the kJ on food labels and databases. It would no longer be necessary to capitalize the calorie because it would have one meaning: the amount of heat (defined as 4,186 J) required to raise the temperature of 1 kg of water from 14.5 to 15.5°C. These actions would eliminate the confusion caused by having two unnecessary words that scientists introduced and should remove from the vocabulary. After all, starting in about 1970, nutrition scientists grudgingly made the transition from Calories to kcal, but the original plan was to abandon all forms of calorie after a short sunset [45,46] That plan could be completed by simply changing the style guides in major journals. The outcome would be the elimination of "calorie confusion" with scientists retaining their joules and the popular press continuing to employ calories in recipes and diet plans, as they have done for a hundred years.

## Abbreviations

OED: Oxford English Dictionary; 

BIPM: Bureau International des Poids et Mesures;

SI: *Systém International des Unites*.

## Appendix

a). Calorie will be capitalized when the context refers to the modern kcal (4.186 kJ). The lower-case calorie will be used when the g-calorie (4.186 J) is meant.

b). In addition to *Le Producteur*, records at Gallica include Favre and Silbermann's article on calorimetry, copies of L.N. Becherelle's *Dictionnaire National *and Adolphe Ganot's *Traite Elementaire de Physiques*.

c). Nicolas Clément married Claude Desormes' daughter and adopted his father-in-law's name. Nicolas Clément and Clément-Desormes are the same person. One reason for confusion is that their paper on the determination of γ in the gas law is often referred to as "the experiment of Clément-Desormes". Note that Nicolas Clément did not spell his first name with an h.

## Supplementary Material

Additional file 1 The Potential Energy of Food. A facsimile of the following historical article: **Atwater, W.O. The Potential Energy of Food. The Chemistry and Economy of Food. III. Century 1887; 34:397–405**. Click here for file
