# Supplementary material for: Does the history of food energy units suggest a solution to "Calorie confusion"?
Source: Nutr J. 2007 Dec 17;6:44. doi: 10.1186/1475-2891-6-44 (PMC2238749; doi:10.1186/1475-2891-6-44)
Supplement: Additional file 1 — The Potential Energy of Food. A facsimile of the following historical article: Atwater, W.O. The Potential Energy of Food. The Chemistry and Economy of Food. III. Century 1887; 34:397–405. [file 1475-2891-6-44-S1.pdf]

Facsimile of:

Atwater, W.O. The Potential Energy of Food. The Chemistry and Economy of Food. III. Century 1887; 34:397-405.

THE POTENTIAL ENERGY OF FOOD.

THE CHEMISTRY AND ECONOMY OF FOOD. III.

"Besides the . . . chemical elements, there is, in the physical world, one agent only, and this is called energy. It may appear, according to circumstances, as motion [heat], chemical affinity, cohesion, electricity, light, magnetism; and from any one of these forms it can be transformed into any of the others."—FR. MOHR.

"I have here a bundle of cotton, which I ignite; it burns and yields a definite amount of heat; precisely that amount of heat was abstracted from the sun, in order to form that bit of cotton; . . . every tree, plant, and flower grows and flourishes by the grace and bounty of the sun. But we cannot stop at vegetable life, for this is the source . . . of all animal life. In the animal body vegetable substances are brought again into contact with their beloved oxygen, and they burn within us as a fire burns in a grate. This is the source of all animal power, . . . all terrestrial power is drawn from the sun."—TYNDALL.

"All are but parts of one stupendous whole,
Whose body Nature is, and God the Soul;
That changed thro' all, and yet in all the same,
. . . . .
Lives thro' all life, extends thro' all extent,
Spreads undivided, operates unspent."—POPE.

WITHOUT doubt the two most fruitful ideas which our century has developed are those of evolution and the conservation of energy. The latter principle was, I believe, first clearly and definitely set forth in 1837,* just half a century ago, by Dr. Mohr, in the words quoted above.

During the years since then, the astronomers and geologists and physicists have been learning and explaining to us how the energy, whose primordial source in our solar system is the sun, warms and lights our planet; how it is stored in coal and petroleum and wood; and how it is transformed into the heat of the furnace, the light of the lamp, the mechanical power of steam, or into electricity and then into light or heat or mechanical power again. The same energy from the sun is stored in the protein and fats and carbohydrates of food, and the physiologists to-day are telling us how it is transmuted into the heat that warms our bodies and into strength for our work and thought. The potential energy of food may appear in still other forms; — in light, in certain animals, in the

"light of the fire-fly lamp,"

for instance, and even as electricity, in the animal body.

During the epidemic of strikes in the spring of 1886, a church was being built in this city (Middletown, Conn.). When the brick walls were partly laid, the hod-carriers struck for higher pay. The master mason, a man of resources, let them go and got a steam-engine in their place. The brick and the mortar which had been carried up the ladders by Hibernian muscle were lifted by engine and windlass. The work which had been done through the consumption of meat and potatoes in the one case, was accomplished by the combustion of coal in the other, but the underlying principle was the same in both. In each case there was conversion of one form of energy into another. The food which the hod-carriers ate, and the coal which was burned under the boiler, each contained a certain amount of potential energy. That of the food reappeared in the contractile power of muscle, that of the coal in the expansive power of steam.

Before the invention of matches, blacksmiths used to start their fires with iron heated by hammering. The heating of the iron was a case of the conversion of one form of energy into another. The muscular energy of the blacksmith's arm was transformed into the mechanical energy of the descending hammer; when the hammer struck, the energy was imparted to the iron, where it was transmuted into heat, and the iron became red-hot.

The energy came from the blacksmith's food.

* In an article in the "Zeitschrift fur Physik und verwandte Wissenschaften," a journal published in Vienna. It is an interesting fact that these fifty years of unprecedentedly active and brilliant research have only confirmed, while explaining in detail, the principle thus laid down by a young and comparatively unknown German chemist. And it is a striking illustration of the lack of appreciation with which new ideas are often received, that Dr. Mohr's article, which contained this great generalization of modern science, was refused by "Poggendorff's Annalen," the leading German journal of physical science, before it was published in the Austrian journal above named. Dr. Mohr, it is true, did not prove the theory experimentally.

Just how all the potential energy of the food is disposed of in the body, experimental science has not yet told us. But it is certain that part of it is converted into heat and part into the mechanical energy exerted by the muscles. Some of it may be transformed into electricity. There is no doubt that intellectual activity, also, is somehow dependent upon the consumption of material which the brain has obtained from the food, but just what substances are consumed to produce brain and nerve force, and how much of each is required for a given quantity of intellectual labor, are questions which the chemist's balance and the respiration-apparatus do not answer.

ENERGY AND THE UNITY OF THE UNIVERSE.

THE introductory chapter of a German treatise on cattle-feeding explains the nebular hypothesis, a procedure which is perfectly rational when we consider that the profitable feeding of cattle is simply the economical management of matter and energy in living organisms, and that the nebular theory helps us, better than anything else, to understand how the forms of matter and of force which we have to do with have come to be what they are.

That the materials which compose this solid globe, the waters on its surface, the atmosphere around it, the things that live upon it, the planets with which it courses round the sun, the sun itself, and all the innumerable hosts of heavenly bodies that make up the material universe, are of common origin, is a doctrine familiar to us all. That in this material universe there are two things, and two things only, matter and energy, has come to be another of the accepted dicta of physical science. And current metaphysics goes a step farther and resolves matter itself into manifestations of energy.

It is this energy which pervades the universe. It comes to us in the light of the farthest star, which, though traveling with almost inconceivable rapidity, requires uncounted years for its journey hither. A reserve supply was accumulated in our sun, untold ages ago, and he has given and is constantly giving it to the earth as heat and light. In the geologic past it has accumulated in subterranean stores of coal, and it is now and all the while being used to build up every plant and animal that lives and grows upon the surface of our earth. The coal and wood we burn, our food, the reserve material of our bodies, are, like the sun, our reservoirs of latent energy.

This energy which, transmuted into the expansive power of steam, impels the ship, draws the railway train, turns the wheels of industry; which, in the telegraph, can "put a girdle round about the earth in forty minutes"; and which so conveniently transports men, their works, and their thoughts from one corner of the world to the other that the nations are all becoming one, is the same which, stored in the grass of the field or in the grain of wheat, gives the ox his strength, the race-horse his swiftness, and man his power of muscle and brain. Such are the grand conceptions which advancing knowledge brings us.

This energy is in the cyclone that devastates the land, as in the cooling zephyr of a summer's eve; it is in the awful rolling of the thunder and in the lightning's flash, as in the rustle of the leaves and the gentle cooing of the dove. It is in the tramping of armed hosts, the roar of artillery, and the carnage of battle, as in the soft caress and tender lullaby with which the mother sings and soothes her babe to sleep.

I often think that the greatest creation of human genius is the medieval cathedral. If this be so, and if the grandest music is that which floats through the cathedral aisles, if the loveliest transformations of the sunbeams are in the dim religious light that enters through its stained glass windows, if the holiest thoughts are those of its worshipers; the power that lifted the stones of the cathedral into their places, the light that reveals its grandeur and its beauty, the thought that planned its architecture and composed its music, the vibrations on which its music floats, the motions and the voices of those who bend

> "en murmurant sous le vent des cantiques
> Comme au souffle du nord un peuple de roseaux,"

and who, in responsive adoration, express the sentiments of its worship, are all, in one way or another, the products of that energy which once existed in space, rested for eons in the central orb of our system, and part of which, coming to us in those things which we designate as food, abides for a time in our own bodies and our own brains, to give us life and power and thought.

Says Professor Tyndall, in speaking of the law of conservation of energy:

> "Waves may change to ripples, and ripples to waves, magnitude may be substituted for number, and number for magnitude, asteroids may aggregate to suns, suns may resolve themselves into floræ and faunæ, and floræ and faunæ melt in air, the flux of power is eternally the same. It rolls in music through the ages, and all terrestrial energy, the manifestations of life, as well as the display of phenomena, are but the modulations of its rhythm."

Nor does he exaggerate, I think, in saying further:

> "Presented rightly to the mind, the discoveries and generalizations of modern science constitute a poem

more sublime than has ever yet been addressed to the intellect and imagination of man. The natural philosopher of to-day may dwell amid conceptions which beggar those of Milton. So great and grand are they, that in the contemplation of them a certain force of character is requisite to preserve us from bewilderment."

But, after all, this statement of a physical law is only the scientific form of the poetic thought expressed in the words of Pope quoted at the beginning of this chapter. Another poet, and one, it seems to me, whose soul was more exquisitely attuned to the harmonies of Nature than any other, has clothed this sentiment in still finer habiliment of words:

"And I have felt
A presence that disturbs me with the joy
Of elevated thoughts: a sense sublime
Of something far more deeply interfused,
Whose dwelling is the light of setting suns,
And the round ocean, and the living air,
And the blue sky, and in the mind of man:
A motion and a spirit, that impels
All thinking things, all objects of all thought,
And rolls through all things."

WORDSWORTH.

What are the relations of this physical energy, whose "flux is eternally the same," to the Supreme Power that "impels all thinking things" and "rolls through all things," and "though changed through all" is "yet in all the same," it is the office of the metaphysician and the theologian rather than the chemist to discuss. But as physicists have found that all the forms of physical energy are really one, and chemists are aspiring to the proof that the different elements of which matter is composed are merely modifications of one primordial form, so I cannot forbear the conception, I might almost say belief, that one day the advance of knowledge will bring men to feel that the ideas thus framed in words by scientist and poet are one, not only with each other, but with the sentiment embodied in the words of an older and grander poet:

"O Lord, how great are thy works! and thy thoughts are very deep. . . . But thou art the same, and thy years shall have no end. . . . Such knowledge is too wonderful for me; it is high, I cannot attain unto it. . . . If I ascend up into heaven, thou are there: if I make my bed in hell, behold, thou art there.

"If I take the wings of the morning, and dwell in the uttermost parts of the sea;

"Even there shall thy hand lead me, and thy right hand shall hold me."

Indeed, unless I wrongly apprehend the tendency of the speculation of our time, it is decidedly in this direction. In a late essay on "Religion, a Retrospect and Prospect,"* Mr. Herbert Spencer tells us that "amid the mysteries which become the more mysterious the more they are thought about, there will remain the one absolute certainty, that we are ever in presence of an Infinite and Eternal Energy, from which all things proceed." Such leaders of thought as Professors Lotze in Germany and Bowne in this country, and many other metaphysicians with them, teach that the things that we call matter are only forms of action of energy and that this energy is God immanent in the universe. And in his most exhilarating lectures on "The Idea of God," Mr. John Fiske says: "Instead of the force which persists let us speak of the Power which is always and everywhere manifested in phenomena. . . The everlasting source of phenomena is none other than the infinite Power that makes for righteousness"; and again: "The infinite and eternal Power that is manifested in every pulsation of the universe is none other than the living God."

In adopting these conceptions, then, which do away with the conflict between science and religion by making them one in origin and spirit; which teach us that even in the use of our daily bread we are linked to the Power whom we are taught to pray to give it to us; which help us to understand that without his knowledge, because without his action, not even the sparrow falls to the ground; and which help us to realize that the plainest and homeliest things that concern the welfare of our fellow-creatures are worthy of our most serious study and our profoundest thought; we are only following the current philosophy of the time.

That we do not think of these things every time we eat our bread and meat is very well, but such things are worth thinking of once in a while. If they were not, life would not be worth living. But I am wandering far afield and must come back to my subject.

AMOUNTS OF POTENTIAL ENERGY IN FOOD-MATERIALS.

MODERN physical science has taught how to measure the potential energy in combustible materials. The apparatus used is called a calorimeter, and the energy is measured by the amount of heat produced in burning the substances with oxygen, the equivalent of the heat in terms of mechanical energy being quite definitely known. The amounts of potential energy in different food-materials have been measured in this way.

Chemists and physiologists have thought for a long while that when the food is consumed in the body it must yield the same quantity of energy as when burned in the calorimeter. In both cases it is burned with oxygen, although the process in the body is far less simple than in the calorimeter. A number of years ago, Professor Frankland, of London, determined the heats of combustion of differ-

* The Nineteenth Century. Vol. XV.

# DIAGRAM IV. POTENTIAL ENERGY OF FOOD.

CALORIES IN THE NUTRIENTS IN ONE POUND OF EACH FOOD-MATERIAL.

Beef, round, rather lean	807
Beef, neck	1108
Beef, sirloin, rather fat	1173
Beef, flank, very fat	2750
Beef, side, well fattened	1463
Mutton, leg	1142
Mutton, shoulder	1281
Mutton, loin (chops)	1755
Mutton, side, well fattened	1906
Smoked ham	1960
Pork, very fat	3452
Flounder	286
Cod	310
Haddock	331
Bluefish	404
Mackerel, rather lean	430
Mackerel, very fat	1026
Mackerel, average	696
Shad	750
Salmon	967
Salt cod	416
Salt mackerel	1364
Smoked herring	1343
Canned salmon	1036
Oysters	229
Hens' eggs	760
Cows' milk	308
Cows' milk, skimmed	176
Cheese, whole milk	2044
Cheese, skimmed milk	1166
Butter	3691
Oleomargarine	3679
Wheat flour	1655
Wheat bread	1278
Rye flour	1614
Beans	1519
Pease	1476
Oatmeal	1830
Corn (maize) meal	1616
Rice	1627
Sugar	1798
Potatoes	427
Sweet potatoes	416
Turnips	139

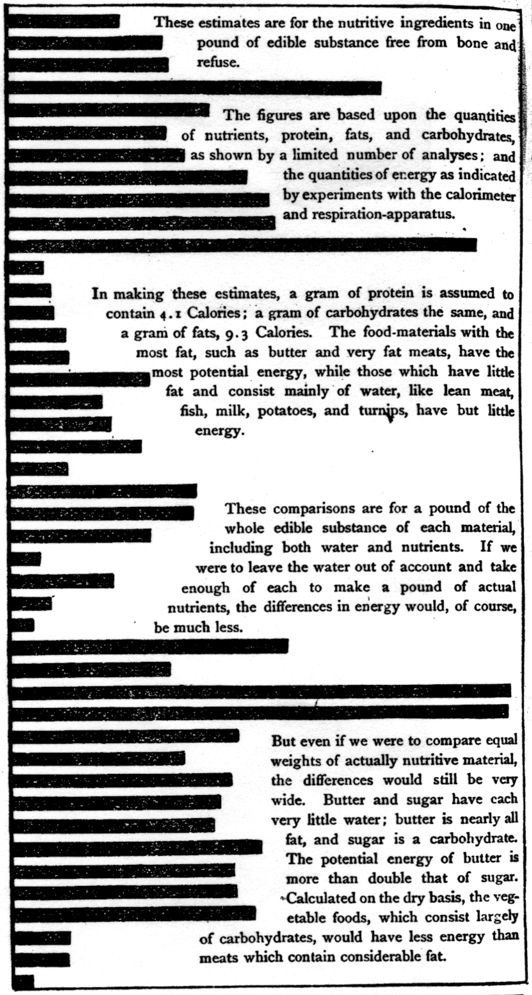

The potential energy represents simply the fuel-value of the food, and hence is only an incomplete measure of its whole nutritive value. Besides serving as fuel, our food has other uses, one of which is, if possible, still more important, namely, that of forming and repairing the tissues of the body, the parts of the machine. This latter work is done by the protein, which has comparatively little potential energy. Protein is the chief nutrient of lean meat and fish. These have, therefore, a high nutritive value, although their energy is comparatively small. (See Diagram III. of first article of this series.)

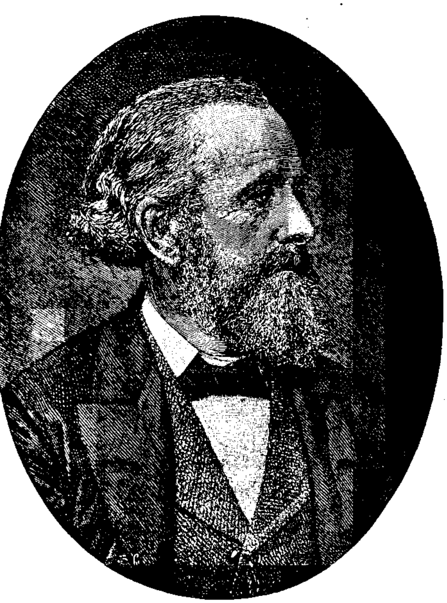

PROFESSOR EDWARD FRANKLAND.

ent food-materials, and his results have since been taken by many chemists and physiologists as the standard for their fuel-values when they are used for food, although with a certain amount of reserve, because of the lack of proof that the heat generated in the calorimeter is an accurate measure of the energy developed by the same materials in the body. The actual demonstration that this is the case, has been reserved for the refinements of later research.

Within a short time past, feeding-trials with animals in the respiration-apparatus have shown the proportions in which the several classes of nutritive ingredients of food do one another's work in serving as fuel in the body, and more extended experiments, with improved forms of the calorimeter, have given very accurate measurements of the amounts of potential energy in the same materials. The respiration experiments have been made with dogs, in the Physiological Institute in Munich, by Dr. Rubner, who has also made an extended series with the calorimeter. The largest number of the experiments with food-materials in the calorimeter, however, have been conducted by Professor Stohmann, of the University of Leipsic, and his assistants. The results of experiments with the respiration-apparatus and with the calorimeter agree with most remarkable closeness. In supplying the body with fuel, the protein, fats, and carbohydrates* replaced on the average, as much heat to keep their bodies warm and energy for the work their muscles had to do, from 243 grams of lean meat (*i. e.*, meat enough to furnish 243 grams of nutritive material after the water had been driven out), as they obtained from 100 grams of fat, while 235 grams of the lean meat, burned to equivalent products in the calorimeter, would yield the same amount of heat as the 100 grams of fat. Considering the great difficulties in experimenting with live animals, these two isodynamic values, 243 by the respiration-apparatus and 235 by the calorimeter, agree very closely indeed. But with starch, the results by the two methods, 232 and 229, are still closer, while with ordinary table sugar and grape sugar they are as good as identical.

Taking our ordinary food-materials as they come, and leaving out slight differences due to the differences in digestibility, etc., Dr. Rubner has made the following general estimate of the amounts of energy in one gram of each of the three principal classes of nutrients. The Calorie, which is the unit commonly employed in these calculations, is the amount of heat which would raise the temperture of a kilogram of water one degree centigrade (or a pound of water 4 degrees Fahrenheit). Instead of this unit of heat we may use a unit of mechanical energy, for instance the foot-ton, which is the force that would lift one ton one foot. One Calorie nearly corresponds to 1.53 foot-tons.

POTENTIAL ENERGY IN NUTRIENTS OF FOOD.

	Calories.	*Foot-tons.*
In one gram of protein	4.1	6.3
In one gram of fats	9.3	14.2
In one gram of carbohydrates	4.1	6.3

These figures mean that when a gram (one twenty-eighth of an ounce) of fat, be it the fat of the food or

* The previous articles of this series have described the different kinds of nutrients of foods. Myosin (lean) of meat, white of egg, casein (curd) of milk, gluten of wheat, etc., are protein compounds. Fat of meat, butter, and oil of corn and wheat are fats. Starch and sugar are carbohydrates.

Since these German researches are very recent and have not yet been made accessible to English readers, I could hardly expect to be excused if I did not give at least an inkling of the details. Here is Dr. Rubner's summary of some of the main results of several long series of experiments, the descriptions of which occupy several hundred pages.

ISODYNAMIC VALUES
FOR ONE HUNDRED PARTS OF FAT.

Nutritive substances, water-free.	*As determined by direct experiments with animals.*	*As determined by calorimeter.*
Myosin	225	213
Lean meat	243	235
Starch	232	229
Cane sugar	234	235
Grape sugar	256	255

The quantities of the several substances, lean meat, myosin (the chief protein compound of lean meat), starch, etc., are those which were found to yield the same amounts of heat when burned in the calorimeter, or to render the same service as fuel when consumed in the body of the animal, as 100 grams of fat. This explanation of the meaning of the expression "isodynamic values for 100 parts of fat" needs a little qualification to make it perfectly correct, but it is as accurate as I can well make it without going into a discussion too abstruse for the pages of a magazine, and it is really accurate enough for our purpose. The figures mean, then, that the dogs in the respiration-apparatus obtained,

each other in almost exact proportion to their heats of combustion. That the living body should thus be proved to use its food with such perfect chemical economy is certainly interesting and important. It is one more fact to add to the long lists that are bringing the functions of life more and more within the domain of ordinary physical and chemical law.

The diagram of "Potential Energy of Food" herewith indicates the amounts of potential energy in different food-materials. The estimates are for one pound of each material; that is to say, for one pound of edible substance, freed from refuse, as for instance, meat without bone or the shell-contents of eggs. It is of course to be understood that the materials vary in composition and that these figures represent averages merely. In fact, both the analyses of the food-materials and the researches upon the potential energy of the nutrients are as yet far too limited in extent to be entirely satisfactory. The diagram is like a map of a new country, based upon the first explorations; in the main correct, but in need of more complete surveys to make it accurate in all its details.

body-fat, is consumed in the body, it will, if its potential energy be all transformed into heat, yield enough to warm a kilogram of water nine and three-tenths degrees of the centigrade thermometer, or, if it be transformed into mechanical energy such as the steam-engine or the muscles use to do their work, it will furnish as much as would raise one ton fourteen and two-tenths feet or fourteen and two-tenths tons one foot. A gram of protein or carbohydrates would yield a little less than half as much energy as a gram of fat. In other words, when we compare the nutrients in respect to their fuel-values, their capacities for yielding heat and mechanical power, an ounce of protein of lean meat or albumen of egg is just about equivalent to an ounce of sugar or starch; and a little over two ounces of either would be required to equal an ounce of the fat of meat or butter or body-fat. The potential energy in the ounce of protein or carbohydrates would, if transformed into heat, suffice to raise the temperature of one hundred and thirteen pounds of water one degree Fahrenheit, while an ounce of fat, if completely burned in the body or in the calorimeter, would yield as much heat as would warm over twice that weight of water one degree.

The calculations of Diagram IV. are based upon the figures just given for the potential energy of each nutrient. The figures used for the quantities of each nutrient in each food-material of Diagram IV. are the same as those on which Diagram III. of the first article of this series is based.

By these calculations, a pound of wheat flour contains as much energy, to be converted into the heat which a laboring man needs to keep his body warm, and muscular strength to do his work, as two pounds of lean beef free from bone, while a pound of very fat pork is equal to over four pounds, and a pound of butter to nearly five pounds of the very lean beef. That is, the quantities of latent energy in lean beef, flour, fat pork, and butter, are to each other as one, two, four, and five.

That these food-materials should differ so greatly in fuel-value may, at first sight, seem a little strange. But when we compare the composition of the very fat and the very lean meat, as shown in Diagram III. of the first article of this series (May CENTURY), the reason becomes clear. The very lean meat consists mostly of water, which has no potential energy, while the very fat meat has extremely little water and is composed mainly of fat, which has more potential energy than any other nutrient. The difference between the very fat meat and the wheat flour is not due so much to difference in their proportions of water, for they have nearly the same, but rather to the fact that flour consists largely of starch, which has relatively little potential energy. Butter and oleomargarine lead all the other materials in their quantities of energy. The fat of butter is slightly inferior in this respect, weight for weight, to the fat of meat, the proportions as found by experiment being as 92 to 94, nearly.

I fear I have not yet made quite clear just what these statements and the figures in the diagram actually mean.

A pound of wheat flour is computed to yield energy equal to 1656 calories or 2534 foot-

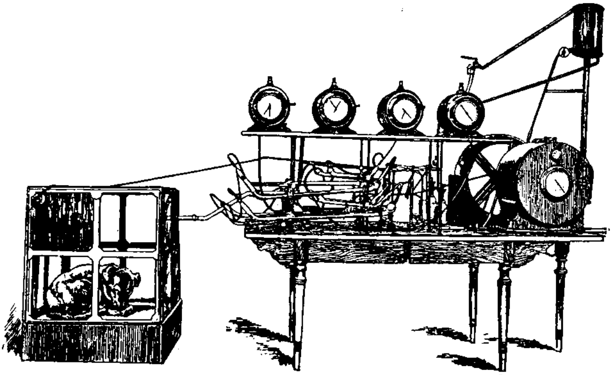

SMALL RESPIRATION-APPARATUS IN THE MUNICH PHYSIOLOGICAL INSTITUTE.

This apparatus, which is, in principle, identical with the large apparatus described in the previous article of this series, was devised by Prof. Voit, and intended for experiments with dogs, geese, and other small animals. Its object is to provide for analysis of the air before and after it has been breathed by the animal, and thus show what products of respiration the animal has imparted to it. The box in which the animal is kept is made of glass. Through this box a constant current of air is drawn and measured by the large meter on the table. A small portion of this, however, is drawn through two of the small meters by which it is measured, and through apparatus on the table by which it is analyzed. Air taken from outside the box is at the same time drawn through the other two small meters and apparatus on the table, and thus measured and analyzed in like manner.

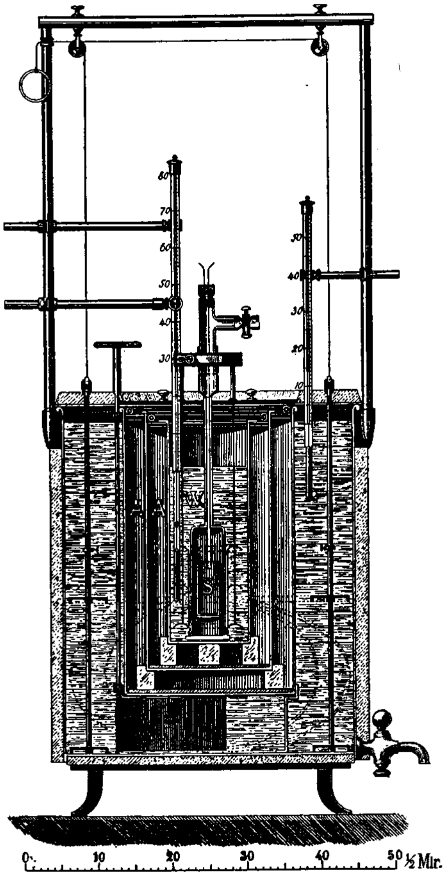

CALORIMETER.

The calorimeter here shown is a late form devised by Prof. Stohmann. Within is a small cylinder, S, in which the substance to be tested is burned, being mixed for this purpose with materials furnishing oxygen. This cylinder is surrounded by a cylindrical cover, and is contained in a larger cylinder, W, holding water. The heat from the burning substance is communicated to the water, and is measured by the rise in temperature as shown by the thermometer. Outside of the cylinder holding the water are two concentric cylinders, A, A, holding air which acts as a non-conductor of the heat. The air-cylinders are surrounded by a larger cylinder containing water, which, in its turn, has a covering of felt, the object being to guard against the influence of changes of temperature of the outer air. The further devices for protecting the interior apparatus from gain or loss of heat, igniting the inner mixture in the inner cylinder and measuring the heat produced by the combustion, need not be described here. The whole apparatus is about eighteen inches wide and a little over three feet high.

tons. But this of course does not mean that if the pound of flour were made into bread, it would enable our blacksmith, if he should eat it, to lift 2534 tons of iron to a height of one foot, or the hod-carrier to carry a ton of bricks to the height of 2534 feet. He could do only a small fraction of this work with his loaf of bread.

Only a very small proportion of the whole energy of the food is made available for external muscular work, such as the hod-carrier's lifting, the blacksmith's hammering, or other manual labor; the most of it is transformed into heat. A considerable quantity is used for the interior work of the body, breathing, keeping the blood in circulation, digestion, etc., but a large part of this is transformed into heat before it leaves the body. Thus the mechanical energy imparted to the blood by the muscles of the heart is changed to heat by the friction of the blood against the vessels through which it circulates. Indeed, there is an old theory that it is this friction that gives the body its heat.

The heat generated in the body, by the combustion of food and otherwise, is continually given off by radiation. With plenty of clothing we can retain enough to keep ourselves warm even in a cold day. Too much clothing may so interfere with radiation as to make us uncomfortably warm. The amount of heat produced in the body is so large that it has been calculated that, if there were no way for it to escape, there would be enough in an average well-fed man to heat his body to the temperature of boiling water in thirty-six hours.

We have a very familiar illustration of the production of heat along with muscular energy in the heating of our bodies when we exercise our muscles. We cannot transform the energy of our food into muscular force without transmuting part of it into heat at the same time. In the body, indeed, as with the steam-engine, but a small part of the energy of the fuel is transformed into mechanical power for work. But the body is more economical in this respect than the best steam-engine; that is to say, it gets more power for work from the same amount of energy in its fuel. It has been estimated that while the most efficient steam-engines cannot get more than one-eighth of the energy of their fuel in the form of mechanical power, the body can get one-fifth. Some calculations, indeed, make a far more favorable showing for the animal as compared with the machine in respect to economy in the use of fuel for work. Professor von Gohren, as the result of elaborate computations, reckons that

A horse may transform			32	per cent.	
An ox	"	"	43	"	"
A man	"	"	53	"	"

of the whole potential energy of his food into energy for mechanical work. More research is needed, however, before entirely satisfactory calculations of this sort can be made.

But to come back to the energy in our hod-carrier's pound of flour. If four-fifths are transformed into heat in his body and only one-fifth into muscular force for work, this would give him 500 foot-tons of muscular energy. But when he climbs the ladder with his hod of bricks he must carry his body and the hod up and down again; the power his muscles use to lift their load is not applied directly but through

a complex system of levers in his limbs, and much of the power is used in other ways; so that the amount of lifting of bricks bears a very small proportion to the total energy of the food.

Just as I am writing this, the last volume of the transactions of the Bavarian Academy of Sciences comes to hand, with a communication from Dr. Rubner, giving account of some new and extremely interesting experiments in this direction. I hope the fact that one object of these articles is to report the latest news from the field of abstract research will excuse at least a brief reference to the main results. The experiments were in continuation of those mentioned above, and, like them, were made with dogs in the respiration-apparatus.

One principle which they bring into clear relief is the remarkable economy with which the animal organism uses its material when the supply is limited, and the positive wastefulness it practices when the food-supply exceeds the demand.

The dogs had very little room to move about inside the apparatus and of course made very little muscular exertion. Hence they needed but little protein to make up for the wear of muscle, and, practically, the main demand of their bodies was for fuel to yield heat to warm their bodies and strength for the very little work their muscles had to do. When they fasted, they consumed the fat and protein from the store in their bodies. How rigidly economical they were in this draft upon their previously accumulated capital was shown in the way that the consumption of fuel was affected by the temperature of the room. The interior temperature of the body remained very nearly the same, at "blood-heat," all the while, as indeed it must, or the dogs would have died. In cold days more heat was radiated from the body than in warm, more was needed to supply its place, and more material was consumed. When the room was warmer the body burned less fuel. And the quantities consumed marked the changes of temperature with a delicacy almost comparable with that of the thermometer.

When the dogs had just food enough to supply their needs they used it with similar economy. In other words, when the income was equal to the necessary expenditure it was used as sparingly as the sums taken from the capital had been. When the food-supply was made larger, part of the extra material was stored in the body as fat and protein, but at the same time the daily consumption increased. That is to say, when their income was more liberal, they laid part of it by, but at the same time allowed their current expenses to increase. It has been found by numerous experiments that when the nutrients are fed in large excess the body may continue for a time to store away part of the extra material, but after it has accumulated a certain amount it refuses to take on more, and the daily consumption equals the supply even when this involves great waste. With the large income, the body continues for a time to add to its capital, but finally it comes to spend as much as it gets, and in so doing practically throws away what it cannot profitably use.

Dr. Rubner's dogs showed, in still another way, their economy of fuel when the supply was limited, and wastefulness when they had more than they needed. The same animal that adjusted its consumption of fuel so accurately to the temperature of the air as long as the amount did not exceed its need, used it with no apparent regard to the temperature, whether warm or cold, as soon as the supply of food exceeded the necessary demand.*

This all seems very simple and natural. So the laws of nature always do when we have discovered and begin to understand them.

* Physiologists have observed that the consumption of fuel in the body sometimes varies with the temperature and sometimes does not, and have been at a loss to explain the apparent discrepancies in their experimental results. These experiments help toward an explanation. But the interesting point is, not simply that the facts are learned, but that they are learned by studying the subject from the standpoint of the potential energy of the food. Previously, the accounts have, so to speak, been drawn up in terms of protein, carbohydrates, and fats, and the balances have been difficult to calculate and still more difficult to explain. But in the experiments of which I have just been speaking, all the figures were reduced to terms of potential energy of the food and body-substance consumed or stored. The results were calculated in Calories, and the balancing of the accounts was thus made simple, and the explanation plain.

Of course I do not mean to say that we have thus suddenly come upon a complete explanation of the whole subject. This is simply an improvement of methods based on clearer understanding of principles and leading to clearer and more accurate results. It is, in short, the old story of clearing up an old mystery by use of a new and rational idea. As such, as well as for stronger reasons, it is of interest.

It is so easy to magnify the importance of any new discovery, and so hard to avoid going too far in drawing inferences from it, that I am inclined to put in another word of caution here. For instance, from the experiments above described one would infer that the food-ingredients yield strength for muscular labor in exact proportion to their heats of combustion. But the dogs in the respiration-apparatus performed no muscular work except that inside their bodies for respiration, keeping the blood in circulation, etc., and though we naturally assume that if they had used their muscles for exterior work, such as running or working a treadmill, the muscular energy yielded by the food would have been likewise equal to its potential energy, and though the other known facts make this assumption entirely probable, the experiments do not absolutely prove it. The production of muscular strength is a problem which is still but partly solved. Still I think it is reasonably safe to say that, in general, the foods that have the most potential energy are the ones that yield, not only the most heat to keep the body warm, but also the most strength for muscular work.

THERE are numerous homely, practical ways in which these principles may be applied. I well remember how the sensible and thrifty New England people among whom my boyhood was spent used to talk about "hearty victuals," and how prevalent were the doctrines that "a hard-working man wants real hearty food," and that "children ought to have hearty food, but not too hearty."

With these eminently orthodox tenets the science of nutrition in its newest developments is in fullest accord. But there always used to be an unsatisfactory vagueness about them. I never could make out exactly what were "hearty" foods, and in just what their heartiness consisted. It has since occurred to me that these words express one of the ideas which the unerring sense and instinct of man have wrought out of his long experience, but have waited for science to put into clear and definite form. The synonym with which our science defines this idea is energy. Hearty foods are those in which there is an abundance of potential energy.

The lumbermen in the Maine forests work intensely in the cold and snows of winter and in the icy water in the spring. To endure the severe labor and cold, they must have food to yield a great deal of heat and strength. Beans and fat pork are staple articles of diet with them, and are used in very large quantities. The beans supply protein to make up for the wear and tear of muscle, and they, and more especially the pork, are very rich in energy to be used for warmth and work.

I cannot vouch for the following, which has just struck my eye in a daily paper, but, if it is true, the workmen were sound in their physiology:

"A lot of woodchoppers who worked for Mr. S—— in H—— stopped work the other day, and sent a spokesman to their employer, who said that the men were satisfied with their wages and most other things, but didn't like 'your fresh meat; that's too fancy, and hain't got strength into it.' Mr. S—— gave them salt pork three times a day, and peace at once resumed its sway."

The use of oily and fatty foods in arctic regions is explained by the great potential energy of fat, a pound of which is equal to over two pounds of protein or starch. I have been greatly surprised to see, on looking into the matter, how commonly and largely the fatter kinds of meat are used by men engaged in very hard labor. Men in training for athletic contests, as oarsmen and foot-ball teams, eat large quantities of meat. I have often queried why so much fat beef is used, and especially why mutton is often recommended in preference to beef for training diet. Both the beef and the mutton are rich in protein, which makes muscle. Mutton has the advantage of containing more fat along with the protein, and hence more potential energy. Perhaps this is another case in which experience has led to a practice, the real grounds for which have later been explained by scientific research.

The Germans have, in their vernacular, hit closer to the principle here explained than we. Their scientific expression for energy is *Kraft*. In their folk-tongue the word for nourishing, strength-giving, is *kräftig*. When, as a newcomer, I first looked for a boarding-place in a German city, I was amused at the recurring assurances from would-be hosts and hostesses, that their fare was *kräftig*. With the abundance that crowns even the humble board at home, I had not learned how much that word and the idea it carried could mean in less favored lands.

W. O. Atwater.

CROOKED JOHN.

THE Von Gravens had once been a great family; but reckless living had ruined them. They were large, handsome men, with blue eyes and a distinguished bearing. No end of stories were told in the valley of their bright sayings and their foolhardy deeds. Some of their observations, I regret to say, were not for ears polite. Colonel Von Graven, who, with his son Harold, was now the only bearer of the name, was understood to have been a lady-killer in his day. When his dignity thawed out over a glass of toddy, he had been known to make allusion to his adventures in that line; and when the judge gave him a dig in the ribs and called him a gay old boy, he did not resent it.

Harold, the colonel's son, was a regular dare-devil. They called him "the girls' Harold," because he had such a taking way with women. Nobody could hold a candle to him on the dancing-floor; and the colonel rubbed his hands and chuckled when he saw him take to love-making as a duck does to water. Ah, yes, he made havoc in the hearts of the girls in those days — mere lad as he was.

Then it was that one fine day in the spring there was a log-jam in the river. The water rose several feet an hour, and there was a roar in the air as of a hundred chariots. Two men had gone to the bottom in trying to break the jam, and it seemed sure death for the third and the fourth. The cataract thundered below, and the yellow foam flew high over the tree-tops. The
